# Inguino-Abdominal Eventration

**Published:** 1916-10

**Authors:** 


**Inguino-Abdominal Eventration.** Mercade, Soc. de Chir., Feb. 23, reports a case of a soldier aged 24, wounded by shrapnel. The intestine did not escape from the wound but was immediately beneath the skin and while easily returned, could not be kept in the abdominal cavity. A month later, the right rectus and the crural arch were found lacking. The sartorius was exposed, cut below its upper third and the inferior end attached to the vastus externus. The upper end was brought in front of the hernial orifice. The muscle of the obliquus externus was sutured to the pubis, pectineus, sheath of the vessels and psoas. A small hernia persists but is sustained by a muscular belt which allows the patient to exercise without discomfort.

